# Determination of phage susceptibility as a clinical diagnostic tool: A routine perspective

**DOI:** 10.3389/fcimb.2022.1000721

**Published:** 2022-09-21

**Authors:** Valéry Daubie, Houssein Chalhoub, Bob Blasdel, Hafid Dahma, Maya Merabishvili, Tea Glonti, Nathalie De Vos, Johan Quintens, Jean-Paul Pirnay, Marie Hallin, Olivier Vandenberg

**Affiliations:** ^1^ Innovation and Business Development Unit, LHUB-ULB, Université Libre de Bruxelles, Brussels, Belgium; ^2^ Department of Microbiology, LHUB-ULB, Université Libre de Bruxelles, Brussels, Belgium; ^3^ Centre for Environmental Health and Occupational Health, School of Public Health, Université Libre de Bruxelles (ULB), Brussels, Belgium; ^4^ R&D department, Vesale Bioscience, Noville-sur-Mehaigne, Belgium; ^5^ Laboratory for Molecular and Cellular Technology, Queen Astrid Military Hospital, Brussels, Belgium; ^6^ Department of Clinical Chemistry, LHUB-ULB, Université Libre de Bruxelles, Brussels, Belgium; ^7^ Division of Infection and Immunity, Faculty of Medical Sciences, University College London, London, United Kingdom

**Keywords:** phage (bacteriophage), susceptibility, clinical microbiology, diagnosis, personalized medicine

## Abstract

As the global burden of disease caused by multidrug resistant bacteria is a major source of concern, credible clinical alternatives to antibiotic therapy, such as personalized phage therapy, are actively explored. Although phage therapy has been used for more than a century, the issue of an easy to implement diagnostic tool for determining phage susceptibility that meets current routine clinical needs is still open. In this Review, we summarize the existing methods used for determining phage activity on bacteria, including the three reference methods: the spot test, the double agar overlay plaque assay, and the Appelmans method. The first two methods rely on the principle of challenging the overnight growth of a lawn of bacteria in an agar matrix to a known relative phage to bacteria concentration and represent good screening tools to determine if the tested phage can be used for a “passive” and or “active” treatment. Beside these methods, several techniques, based on “real-time” growth kinetics assays (GKA) have been developed or are under development. They all monitor the growth of clinical isolates in the presence of phages, but use various detection methods, from classical optical density to more sophisticated techniques such as computer-assisted imagery, flow-cytometry, quantitative real-time polymerase chain reaction (qPCR) or metabolic indicators. Practical considerations as well as information provided about phage activity are reviewed for each technique. Finally, we also discuss the analytical and interpretative requirements for the implementation of a phage susceptibility testing tool in routine clinical microbiology.

## Introduction

Bacteriophages or phages are the most abundant biological entities on Earth (estimated at 10^31^) (Fuhrman, 1999; Fernández et al., 2019). Phages are natural predators of bacteria that employ the cellular machinery of their host for replication (Adesanya et al., 2020). A productive infection starts with phage adsorption to the bacterial cell surface, followed by injection of the viral genetic material into the cytoplasm. Obligately lytic phages then hijack or replace host cell genomic replication, transcription and translation machineries for the production of new virions. The last step involves the release of these newly produced virions (Maura and Debarbieux, 2011). Viral progeny can then continue the cycle by diffusing to new permissive bacterial hosts (Abedon et al., 2011). These cycles, conducted on a global scale, account for 20-50% of bacterial mortality (Wommack and Colwell, 2000).

Bacterial and phage populations co-exist through dynamic evolution, and competition among them is one of the major forces driving the evolution of both types of organisms (Rodriguez-Brito et al., 2010). New phage functions are constantly selected during evolution and constitute a promising resource for potential therapeutic application (Schoenfeld et al., 2010), especially in human bacterial infections. Indeed, multidrug resistant (MDR) bacteria are an increasing source of concern (Maura and Debarbieux, 2011), as the pipeline of drugs targeting such bacteria is almost empty (Kumarasamy et al., 2010).

The use of phages in human therapy began over a century ago, in 1919, but was overshadowed a generation later in Western countries when antibiotics were taken into use during the Second World War (Gordillo Altamirano and Barr, 2019). Seeing phage therapy as an alternative to Western advances in antibiotic development and production, the former Soviet Union, its satellite states and allies further developed it as a parallel method of antimicrobial control (Abedon et al., 2011; Pires et al., 2020). As antibiotic resistance is now perceived as the current biggest threat to public health (Malik and Bhattacharyya, 2019), the medical application of phages is being reconsidered in Western countries (Pires et al., 2020). Studies in Eastern Europe have provided some evidence of phage efficiency in the treatment of certain infections (Sulakvelidze et al., 2001; Abedon et al., 2011; Międzybrodzki et al., 2012; Kincaid, 2019), such as skin or wound infection, pleural infection and bacterial dysentery (for a systematic review of phage therapy safety and efficacy in difficult-to-treat infections see Uyttebroek et al. (2022)). However, clear efficacy data from randomized controlled clinical trials remain scarce (Uyttebroek et al., 2022). Despite abundant and promising pre-clinical work, randomized controlled trials (RCTs) using fixed cocktails of phages, whether or not produced according to Good Manufacturing Practice (GMP), have repeatedly failed to show the expected efficacy (Sarker et al., 2016; Jault et al., 2019; Leitner et al., 2021). The premise of these cocktails, which are formulated following extensive pre-clinical screening against large bacterial collections, is to infect broad proportions of clinical strains of a given pathogen. Administrated to patients while waiting for confirmation of specific *in vitro* activity, they often either failed to be effective against the patient’s strain, or were administered at insufficient (much lower than expected) concentrations due to compromised stability (Pirnay et al., 2011; Pirnay et al., 2018; Rohde et al., 2018; Huang et al., 2019). This has led many to propose the “personalized” model, where the cocktail is formulated to contain phages that have been determined to be active against the patient’s specific clinical isolate, as a more credible path towards efficacious phage therapy (Pirnay et al., 2011; Pirnay et al., 2018; Rohde et al., 2018).

Due to the high host-specificity of phages, the search for a phage that lyses a particular bacterial strain often requires the screening of large phage collections (Pires et al., 2020). While numerous studies on the safety and efficacy of phage preparations are currently being conducted in animal models as well as in humans, no rapid laboratory test has been developed so far that can be implemented to evaluate the susceptibility of a bacterial strain to phages in a “routine” clinical microbiology laboratory setting. Yet, in cases of acute infection, the timely administration of an active treatment is crucial. Therefore, an appropriate diagnostic method for determining phage susceptibility in a clinically relevant time frame is a prerequisite for the routine use of personalized phage therapy.

To consider phage therapy as a credible 21^st^ century answer or addition to failing antibiotics, developing appropriate reliable testing methods that reach at least the same turn-around time as current antibiotic susceptibility testing is now pressing. We review here the methods available to assess the efficacy of a phage library against a specific bacterial strain focusing on their strengths and weaknesses: well-proven ‘historical’ methods, such as the double agar overlay plaque assay, the spot test, and the Appelmans method; but also more emerging techniques are addressed. Practical considerations from our daily experience in a clinical microbiology laboratory and unmet needs for testing and reporting phage activity are also discussed.

## Current methods for bacteriophage activity testing

The life cycle of an obligately lytic tailed phage can be disambiguated into the following sequential steps: virion interaction and adhesion to the bacterial cell (adsorption period), nucleic acid translocation, latent period, and progeny release (Hyman and Abedon, 2009). The duration of these steps, together with the number of phage progeny produced (phage burst size), are parameters that influence a phage’s activity against a bacterial strain. Furthermore, a phage can kill a bacterial cell without being able to replicate at its expense (without releasing progeny), as phenomena such as “abortive infection” or “lysis from without” can occur (Hyman and Abedon, 2009; Abedon et al., 2011).

In addition, bacterial phage resistance mechanisms may act at different stages of the infection: at the “adsorption” stage (loss or absence of receptor, physical barriers), leading to impaired adsorption of the phage to the bacterium; or later on (phage-genome uptake blocks, restriction-modification, CRISPR…), preventing the release of infectious phage particles from the infected cell (Khan Mirzaei and Nilsson, 2015). As all these parameters can affect the clinical success of phage therapy, the extent to which each testing method is able to detect and/or estimate them is of importance. The double agar overlay plaque assay and the spot test are both widely considered as reference methods and both rely on the principle of challenging the overnight growth of a lawn of bacteria in an agar matrix to a known concentration of phage (Carlson, 2005).

### Double agar overlay plaque assay

**Figure 1:**
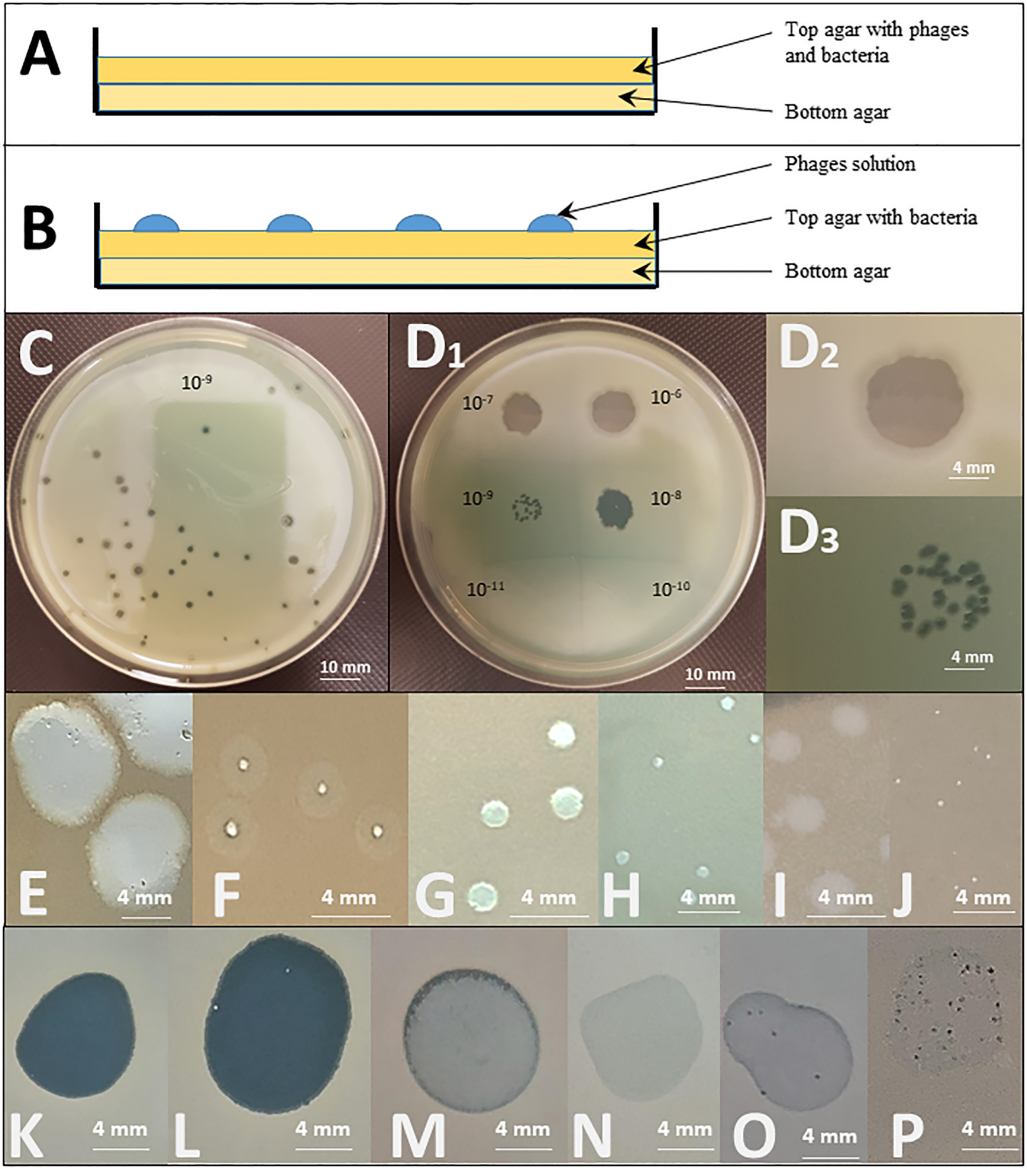
Spot test and double agar overlay plaque assays. Top: Graphical representation of the different layers deposited in a Petri dish for **(A)** a double agar overlay plaque assay and **(B)** a spot test. Middle: Illustration of the testing of the effect of a PEV2 phage suspension (~10^11^pfu/ml) on a *Pseudomonas aeruginosa* isolate by **(C)** a double agar overlay plaque assay (the dilution illustrated here is 10^-9^) and **(D)** a spot test using ten-fold serial dilutions of the phage (10^-6^ to 10^-11^ as indicated). D2: Enlargement of the dilution 10^-6^ spot showing a completely clear (non-turbid) spot indicating that phages killed the tested bacteria. D3: The 10^-9^ dilution spot leads to distinguished plaques. In both **(C)** and D3, the titration of phage, expressed in PFU/mL, can be determined by counting the number of plaques, reported to the concentration and the volume of the phage suspension engaged. In the case illustrated in C, approximately 41 plaques are observed for a plaque assay performed with 100µL of the 10^-9^ dilution. The titer is thus calculated as follows: T = 41 pfu/(10^-1^mL*10^-9^) = 41*10^10^ pfu/mL Bottom: Examples of different forms and shapes that can be observed in double agar overlay plaque assays [**(E–J)** – white background] and spot tests [**(K–P)** - black background] using 3 different phages (LUZ19, 14/1 and PEV2) on 6 different *P. aeruginosa* isolates. Plaques size is influenced by multiple phage’s intrinsic factors such as its size, latency period and burst size. **(E)** large plaques with halo indicating *in situ* phage propagation and amplification; **(F)** clear intermediate to small plaques with halo, **(G)** Intermediate clear plaques. I: turbid plaques. In both **(E, F)**, the clear zones in the plaques indicate *in situ* phage replication, the turbid halo around the clear zone indicates that the phage replication in this zone does not lyse all the bacterial cells, but slows their growth noticeably enough that you can distinguish the slight disturbance on the solid medium. If this happens with high enough frequency all the plaque will look turbid, as observed in **(I)**; **(H, J)** clear small and tiny plaques indicating *in situ* lytic phage replication and propagation. **(K)** clear lytic spot, indicating phage killing. *In situ* amplification of phages might occur as well, but is not detected by the method; **(L)** clear spot with few re-growing colonies suggesting pre-existing resistance or *in vitro* occurring of resistance mechanism; **(M)** turbid spot, and **(N)** slight disturbance of the lawn (very faint halo). Both **(M, N)** are indicative of abortive infection (limited phage propagation); **(O)** several plaques with slight disturbance to lawn, and **(P)** numerous clear to turbid plaques with slight disturbance to lawn. Both **(O, P)** might indicate the presence of a temperate phage in the tested bacterial population.

Originally developed by André Gratia (Gratia, 2000) and formalized by Mark Hancock Adams (Adams, 1959), the double agar overlay plaque assay is a quantitative method on solid medium where a densely growing culture of bacteria is exposed to multiple phage dilutions, using one Petri dish per phage dilution. The relative phage to bacteria concentration is known as multiplicity of infection (MOI) input. Phage preparations are mixed with the bacterial strain in a molten 0.6% agar matrix and dispersed evenly onto solid 1.5% agar medium (Kropinski et al., 2009) (**Figure 1B**). If the phage is capable of propagating on the bacterial strain, the replication-lysis-infection cycle of the phage starts and propagates, being constrained to the surrounding agar gel area. After overnight incubation, the host bacterium forms a lawn on the solid medium, except where infectious phage particles have lysed bacterial cells, propagating outward from an infectious centre. This forms what is known as a “plaque”, a zone without bacterial growth caused by the propagation of one phage particle.

A visible plaque is a complex emergent phenomenon that reflects several critical properties of a phage as it relates to a particular bacterial strain. Indeed, plaque assay demonstrates that not only does the phage kill a bacterial isolate, but that it successfully adsorbs to the relevant bacterium, produces and releases phage progeny (productivity) to infect more cells (propagation). Thus, the formation of a true plaque demonstrates that the phage is not only able to undergo all steps of the lytic cycle, but that the cycle is able to continue in a self-supplying way. The titration of the specific activity of the phage preparation against the bacterium tested is also possible: as the phage suspension mixed with the bacteria is spread out over the complete Petri dish, the number of plaques per plate can be counted as “plaque forming units” (PFU) and reported to the volume of phage dilution added, to end with the titre of the starting suspension expressed in PFU/mL (see **Figure 1C**). Furthermore, the relative activity of a phage against a specific clinical bacterial strain can also be expressed as compared to its activity against the reference host strain (titred in parallel through this method). This is expressed as its “Efficiency of Plating” (EOP):


$$EOP=\frac{{\left(PFU/mL\right)}_{tested\;bacterium}}{{\left(PFU/mL\right)}_{reference\;host\;bacterium}}$$

EOP can either be expressed as a positive real number or as a percentage. While, by definition, the EOP of the phage against its reference host strain is 1 (or 100%), its EOP against a test bacterial strain can either be below or above 1, following its activity on that particular strain. As an example, some authors even use to categorize phage activity against test bacterial strains as high (0.1< EOP), moderate (0.005< EOP< 0.099), low (EOP< 0.005), or inexistent (no plaques detected) (Green et al., 2017). Besides EOP, the double agar overlay plaque assay allows to study the plaques’ morphology and size which are also of importance when evaluating phage activity as they can vary according to the phage’s size, its latency period, burst size and diffusion rate (**Figures 1E–P**).

From a clinical point of view, double agar overlay plaque assay indexes if a phage can be used as an “active” treatment (see Glossary), which is, by definition, dependent on both bacterial lysis and *in situ* phage proliferation (Abedon, 2017).

### Spot test

A pure culture of a bacterial strain is diluted in a molten agar matrix (top agar 0.3% to 0.8%) and dispersed evenly in a Petri dish onto solid agar medium (bottom agar 1.5%) so that it can grow into a bacterial lawn. After solidification of the top agar matrix, a small defined volume (usually 10 µL) of a known dilution of phage suspension, is dropped on the top agar (**Figure 1B**).

Drop deposition is typically repeated on the same dish, either to test the sensitivity of the bacterial strain to an array of different phages or to multiple successive dilutions of the same phage, to determine a titre. The titre is determined by counting the number of individual plaques within the area of spots at terminal dilutions that show activity (see **Figure 1D**). Of note, when using high phage concentrations, ‘false positive’ results can occur as phenomena such as lysis from without or abortive infection can produce a unique large clear zone (Xie et al., 2018). Consequently, from a clinical point of view, a unique large clear zone only suggests, as a proximate outcome, that the phage preparation can at least be used as what is called a “passive” treatment, where only cell killing needs to occur, with or without complete phage replication cycle (Abedon, 2017). Some authors underline the importance of using low titres of phage when investigating its capacity to both lyse the host strain and produce new virions (de Melo et al., 2019). Other consider that the spot test should only be used as qualitative activity detection method that has to be confirmed by double agar overlay plaque assay (Glonti and Pirnay, 2022).

### Appelmans method

This method was first developed in the 1920s by René Appelmans for phage titration in liquid media (Appelmans, 1921). Of importance, the term refers to a multiplicity of variations of the process by which a phage (or a cocktail of phages) is grown iteratively in liquid cultures of one or several bacterial host(s) to increase its activity, inhibit the emergence of phage-resistant bacterial mutants, extend its host range (*in vitro* directed evolution of phages), or assess the stability of its activity (Mapes et al., 2016; Burrowes et al., 2019). Schematically, in its use as a diagnostic technique, the Appelmans method monitors the competition of a phage and a bacterial strain in a liquid culture medium over time (either in series of tubes or in 96 well plates). After overnight incubation, the culture is watched for visible lysis, indicating productive infection. Then over a three-day time span, the appearance of “re-growth” by resistant mutants is repeatedly watched for. The longer the medium stays clear, the more active the phage is against that strain.

### Other methods

The three historical methods mentioned above possess the great advantage of not requiring sophisticated or expensive material. However, these elaborate techniques require long hands-on times, overnight incubation and highly skilled and well-trained operators. Furthermore, as both plaque and spots can show a wide range of size, shape and clearance (**Figures 1E–P**), the results are interpretative, thus subject to interpersonal variations and subjectivity.

Numerous methods have recently been developed to overcome these drawbacks. These new methods mostly rely on the same fundamental principle as historical reference methods, which is to challenge bacterial growth with phages, either on solid agar or (for most of them) in liquid media. The innovations lie in [1] the automation (of the whole process and/or the detection step) reducing time and resource-consumption and/or [2] the use of new, more sophisticated detection techniques, allowing to move from qualitative “end point” results (naked eye visual observation) to quantitative measurements that, if made iteratively, enable “real-time” results, mathematical-based data analysis and long-term saving of results.

For instance the use automatable pipetting robots and microtiter plates, combined to digital iterative measures applied to a liquid-based Applemans technique allows to generate dynamic growth curves, making possible to accurately calculate bacterial growth reduction. These emerging techniques based on “real-time” growth kinetics all monitor the growth of clinical isolates in the presence of phages but use various detection methods (**Table 1**), going from classical optical density (OD) to more sophisticated techniques that dynamically index either bacterial cells viability status (live, damaged and dead cells) such as flow-cytometry -based techniques or specifically target living bacterial cells, such as metabolic indicators [in particular Tetrazolium Dye (TD) - for a comprehensive review, see (Braissant et al., 2020), or quantitative real-time polymerase chain reaction (qPCR)]. OD and TD growth kinetics assays (GKA) both possess the double advantage of being potentially high-throughput and to explore both logarithmic and stationary bacterial growth phases. But, while bacterial debris caused by lysis may contribute to underestimate the phage lytic activity using the OD values, the use of TD, measuring respiration, allows to exclude the dead cells and other debris from the measurement. Furthermore, TD assays based on the OmniLog™ system also allow for more complex analyses, such as phage-antibiotic synergy (Henry et al., 2012). This is nowadays a substantial asset, as experts are increasingly agreeing that phages will more probably be used in combination with antibiotics rather than totally replace those (Glonti and Pirnay, 2022).

**Table 1:** Features overview of several methods currently used for phage susceptibility determination.

Methods			Principles			Flexibility					Equipment- infrastructure			Ref.
			Short description	EP/RT	TAT (h)*	Hands-on	Detection	Throughput	Need of a ref. strain	Custom range (phages/bacteria)	Specific equipment	Specific reagents/consumables	Expertise	
**Agar matrix culture**	Ref.	Spot test	Challenging in a Petri dish the growth in the agar matrix of a bacterial lawn with drops of phage suspension. After overnight incubation, a lysis zone (spot) is visible in areas corresponding to the drop sites of active phages.	EP	18	++ (Manual)	NE, Automation possible	Medium	No	Yes/Yes	No	No	+++	(Khan Mirzaei and Nilsson, 2015)
	Ref.	Overlay plaque assay	Growing multiple preparations composed of a same titration of a bacterial host with different phage dilutions, in a molten agar matrix and dispersed evenly onto solid agar (one Petri dish per preparation). After overnight incubation, a “plaque” is a zone where bacterial growth was prevented by the effect of the phage.	EP	18	+++ (Manual)	NE, Automation possible	Low	Yes (EOP calculation)	Yes/Yes	No	No	+++	(Khan Mirzaei and Nilsson, 2015; Abedon, 2017; Green et al., 2017)
	GKA using imaging of plaque growth		Counting and monitoring of plaque growth (see plaque assay) kinetics of a plaque assay using computer-assisted lensless device imaging.	RT	3- 18	++ (Manual)	Automated	Low	No	Yes/Yes	Yes	No	+	(Perlemoine et al., 2021)
**Liquid culture**	Ref.	Appelmans	Monitoring the competition of a phage and a bacterial strain in a liquid culture medium over time (either in series of tubes or in 96 well plates). Over a three days’ incubation, the culture is first watched for visible lysis, then for appearance of “re-growth” by resistant mutants.	EP	18-72	++ (Manual/Semi-automated)	NE, Automation possible	Medium/high	No	Yes/Yes	No	No	+	(Appelmans, 1921)
	GKA using PMA - qPCR		Enumeration of bacterial cells surviving phage exposure in a liquid culture using propidium monoazide, a microbial membrane-impermeable dye that inhibits amplification of extracellular DNA and DNA within dead or membrane-compromised cells prior to amplification by qPCR using bacterial specific primers	RT	5	++ (Manual/Semi-automated)	Automated	Medium/high	No	Yes/No	Yes	Yes	++	(Liu et al., 2014)
	GKA using Flow cytometry		Enumeration and viability status evaluation (live, damaged and dead cells) of bacterial cells exposed to phages in a liquid culture, based on light scattering and fluorescence using live/dead dyes	EP/RT	1-2	++ (Manual)	Automated	Medium	No	Yes/Yes	Yes	Yes	++	(Low et al., 2020; Melo et al., 2022)
	GKA using Optical density		Optical density real-time measurement of a liquid bacterial culture in the presence of phages in a 96 well plate using an automated plate reader optical density over time in an incubating, aerated environment.	RT	up to 12	+ (Automated)	Automated	High	No	Yes/Yes	No	No	+	(Xie et al., 2018)
	GKA using Tetrazolium Dye		Colorimetric real-time measurement of a liquid bacterial culture in the presence of phages in a 96 well plate using an automated plate reader. The signal is produced by metabolically active cells reducing a tetrazolium dye.											(Xie et al., 2018; Duplessis and Biswas, 2020)

EP, EndPoint; EOP, Efficiency of Plating; qPCR, quantitative real-time Polymerase Chain Reaction; Ref., Reference; RT, RealTime; NE, Naked Eye; GKA, Growth Kinetics Assay; PMA, Propidium Monoazide; TAT, Turn Around Time; *starting from pure bacterial culture.

By contrast, instead of monitoring bacterial growth, computer-assisted imagery techniques applied to solid agar plaque assay allow to index the number and the growth kinetics of viral plaque formation in a much shorter turnaround time (TAT) than the traditional plaque assays. Moreover, it allows the detection of phage-resistant bacterial microcolonies inside the boundaries of plaques and can thus track phage resistance, as bacterial re-growth of initially phage-sensitive bacteria still requires phage plaque and bacterial colony formation on agar (Perlemoine et al., 2021; Glonti and Pirnay, 2022).

However, as they usually necessitate large and/or expensive devices, all these technically faster and/or less hands-on time consuming approaches are often restricted to reference laboratories. This implies a subsequent increase of turnaround time due to the delay needed to ship clinical isolates to the core facilities where these tests are implemented. Most important is the total lack of standardization between these methods, let alone the absence of standardized “breakpoints” to determine if a phage has adequate activity to be clinically used. As learned with traditional antibiotics, such technical and interpretation standards are the cornerstone of an efficient antibacterial activity evaluation.

In summary, while more advanced technologies and techniques are emerging, the overlay plaque assay continues to represent the gold standard in determining the susceptibility of bacterial strains to phage, as it explores the phage-host interaction comprehensively (covering multiple rounds of infection, lysis, and release of progeny) in a relatively short (24-48 h) time span.

## Laboratory requirement

Many of the logistical challenges currently faced regarding the development of new diagnostic tests in support of phage therapy have been gradually overcome in the past for Antimicrobial Susceptibility Testing (AST), which currently directs antibiotic therapy: protocols and infrastructure exist to collect patient samples, in a variety of indications, and to generate pure cultures of the causative bacterial agents. While classical AST procedures still require up to 18 hour of additional incubation after the production of a pure culture isolate, emerging techniques tend to reach an actionable antibiotic susceptibility profile within one to two hours, starting from a pure bacterial culture. For phage susceptibility, the TAT still remains a logistic challenge. In order to turn personalised phage therapy into a clinically relevant alternative or addition to antibiotic therapy, any “Phage Susceptibility Testing” (PST) method should at least meet both the TATs and the technical feasibility of existing AST techniques. Ideally, they should also fit into existing clinical sampling protocols and analytical flows.

As many as possible of the following specifications should also be met:

- high sensitivity and specificity
- high throughput
- random access (as opposed to bulk or batch requirement)
- inexpensive disposables or equipment requirement
- user friendliness (as opposed to the need of highly skilled and trained technical staff)
- low TAT (definitely not longer than the time needed for AST)
- clear-cut standardized results (as opposed to interpretative results)

In addition, unequivocal results and easiness of understanding by clinicians are cornerstones for a broad use of phage therapy, as it would ease the translation into standardized clinical actions. A categorical format such as the “sensitive, intermediate, resistant” (SIR) classification currently used to report antibiotic activity against bacteria, would be ideal. Green et al., for example, proposed a four categories classification (strong, intermediate, weak killer and no killing) based on EOP (Green et al., 2017).

## Regulatory considerations

When developing new medical devices for *in vitro* diagnostic use in Europe, one needs to comply with Regulation (EU) 2017/746 of the European Parliament and of the Council of 5 April 2017 on *in vitro* diagnostic medical devices (IVDR) and to fulfil its requirements (Verbeken et al., 2007; EUR-Lex, 2017). A CE-label obtained through a certification procedure with a Notified Body is necessary to sell the device on the European market, as this device will probably fall under class C. As for other diagnostic tools assessing antimicrobial susceptibility, a diagnostic test for directing phage therapy would be considered as a medical device for *in-vitro* diagnostic use with the intended purpose of testing the effectiveness of a specific treatment; the specific treatment being here the phage therapy (European Commission - Public Health, n.d). These IVDR requirements include clinical performance studies validating the ability of the device to correlate with *in-vivo* observations.

## Conclusions

Easy access to phage therapy is a challenge that we urgently need to overcome in the light of the increasing burden of MDR bacterial infections. Due to their relatively long TATs and a lack of standardisation, current phage susceptibility tests are not suitable for routine use in hospital clinical microbiology laboratories. The development of new user-friendly technologies providing clinically high-throughput and “easy-to-interpret” phage susceptibility testing in a timely manner is essential for a widespread clinical implementation of phage therapy.

## Author contributions

VD, HC, BB, HD, MM, TG, NV, JQ, JP, MH, OV: drafting the article or revising it critically for important intellectual content. All authors contributed to the article and approved the submitted version.

## Funding

This work was supported by a research grant from the Walloon Region (Win2Wal project n°, 1910081), Belgium. The authors would also thank iris-Research Fund managed by the King Baudouin Foundation. Any opinions, findings, and conclusions or recommendations expressed here are, however, those of the authors and do not necessarily reflect Walloon Region, iris-research or Vesale Bioscience’ views. Vesale Bioscience provided salary support for authors BB and JQ.

## Conflict of interest

The handling editor GR declared a past co-authorship with the author(s) MM, JP and BB.

## Publisher’s note

All claims expressed in this article are solely those of the authors and do not necessarily represent those of their affiliated organizations, or those of the publisher, the editors and the reviewers. Any product that may be evaluated in this article, or claim that may be made by its manufacturer, is not guaranteed or endorsed by the publisher.

## Glossary

**Table:**

***Abortive infection***	phage infection that ends with both bacterial and phage death.
***Active treatment***	phage therapy achieving both lysis and “actively” produce new virions for eliminating the target bacteria, *as opposed to a passive treatment* where phages kill bacteria but do not replicate themselves to achieve the elimination of the target bacteria.
***Burst size***	average number of phage particles produced per lysed infected bacterium
***Latent period***	duration of a phage infection, begins with attachment and nucleic acid translocation into the bacterial cell, and (for obligately lytic phages) ends with bacterial lysis.
***Lysis from without***	bacterial lysis occurring at the adsorption stage, resulting from the disruption of the cell wall either due to multiple phage adsorption or to the action of phage lysins. This lysis -as opposed to the lysis occurring at the end of a phage life cycle (lysis from within)- does not release progeny, and can be considered as a form of abortive excessive multiple infection.
***Multiplicity of infection***	ratio of infecting phages to bacterial target cells. When referring to a suspension of bacterial cells inoculated with phages particles, the added ratio can be referred to as “MOI_input_”, whereas the terms “MOI_actual_ ” or “multiplicity of adsorption” refer to the actual adsorbed ratio, counting only those phages that have attached to and then infected bacteria
***Obligately lytic (as opposed to temperate or chronic) phage***	Obligately lytic phages exclusively attempt to take over the machinery of the cell, lyse it, and release new phage particles.
***Plaque***	small area without bacterial growth, observed in mixed suspension of bacteria and phages incubated in agar gel. It indicates that phages have replicated, lysed the bacteria and propagated in this area.
***Productive infection***	a phage infection that directly leads to the maturation and release of phage progeny
***Reference host strain***	usually the bacterial strain from which the phage has first been isolated or is produced.
***Resistant bacterium***	any bacterial strain that a particular phage is unable to replicate in
***Spot***	growth inhibition observed in a zone of a bacterial lawn where a phage suspension was dropped before incubation (spot test). A spot formation indicates the phage suspension was able to inhibit the bacterial lawn formation rather than phage infection and/or adsorption had indeed occurred.
***Temperate phages***	phages with the capacity to silence host lethal genes and persist in their host cell incorporating their nucleic acid into the hosts’ chromosome.
